# Characterization of sexual dimorphism in ANGPTL4 levels and function

**DOI:** 10.1016/j.jlr.2024.100526

**Published:** 2024-02-29

**Authors:** Mingjuan Deng, Sander Kersten

**Affiliations:** 1 Nutrition, Metabolism, and Genomics group, Division of Human Nutrition and Health, Wageningen University, Wageningen, The Netherlands; 2 Division of Nutritional Sciences, Cornell University, Ithaca, NY, USA

**Keywords:** lipase/lipoprotein, liver, adipose tissue, triglycerides, ANGPTL4, ANGPTL8, sexual dimorphism

## Abstract

ANGPTL4 is an attractive pharmacological target for lowering plasma triglycerides and cardiovascular risk. Since most preclinical studies on ANGPTL4 were performed in male mice, little is known about sexual dimorphism in ANGPTL4 regulation and function. Here, we aimed to study potential sexual dimorphism in ANGPTL4 mRNA and protein levels and ANGPTL4 function. Additionally, we performed exploratory studies on the function of ANGPTL4 in the liver during fasting using *Angptl4*-transgenic and *Angptl4*−/− mice. Compared to female mice, male mice showed higher hepatic and adipose ANGPTL4 mRNA and protein levels, as well as a more pronounced effect of genetic ANGPTL4 modulation on plasma lipids. By contrast, very limited sexual dimorphism in ANGPTL4 levels was observed in human liver and adipose tissue. In human and mouse adipose tissue, ANGPTL8 mRNA and/or protein levels were significantly higher in females than males. Adipose LPL protein levels were higher in female than male *Angptl4*−/− mice, which was abolished by ANGPTL4 (over) expression. At the human genetic level, the ANGPTL4 E40K loss-of-function variant was associated with similar plasma triglyceride reductions in women and men. Finally, ANGPTL4 ablation in fasted mice was associated with changes in hepatic gene expression consistent with PPARα activation. In conclusion, the levels of ANGPTL4 and the magnitude of the effect of ANGPTL4 on plasma lipids exhibit sexual dimorphism. Nonetheless, inactivation of ANGPTL4 should confer a similar metabolic benefit in women and men. Expression levels of ANGPTL8 in human and mouse adipose tissue are highly sexually dimorphic, showing higher levels in females than males.

Triglycerides (TG) are present in blood plasma as part of TG-rich lipoproteins, consisting of exogenous chylomicrons and endogenous VLDLs (1). Mounting evidence suggests that elevated fasting and post-prandial plasma TG levels are a causal and independent risk factor for coronary artery disease (CAD), likely via their direct association with remnant cholesterol as the causal factor and/or by exerting a proinflammatory effect on macrophages (2). Maintaining optimal plasma TG levels has gained widespread recognition as a promising strategy to reduce the risk of CAD (3, 4). Consequently, pharmaceutical companies are investing in drugs that aim to reduce plasma TG levels.

Women are well known to have lower fasting plasma TG levels than men at any given degree of adiposity (5, 6). Women also exhibit lower postprandial TG excursions (7). So far, it is not fully clear why. It has been reported that plasma VLDL-TG concentrations are determined primarily by VLDL-TG secretion in men and by the VLDL-TG clearance rate in women (6). The adipose tissue plays a key role in plasma TG homeostasis by regulating plasma TG clearance and subsequent TG storage (8). It has been shown that the proportion of plasma and dietary fatty acids stored in subcutaneous adipose tissue is greater in women than men, while no differences were observed in the liver, muscle, and visceral fat (7). In addition, supplementing an oral lipid load with carbohydrates decreased postprandial lipemia in women but not in men, suggesting that the sex differences in postprandial lipid metabolism may in part be due to differences in the hormonal milieu (7, 8).

The hydrolysis of plasma TG in adipose tissue, heart, and skeletal muscle is catalyzed by the enzyme LPL (9). LPL is produced in adipocytes and (cardio)myocytes and is transported by the endothelium-derived protein GPIHBP1 to the capillary surface, where it acts to hydrolyze plasma TG (10, 11). In line with its rate-limiting role in TG uptake, mutations in LPL are associated with severe hypertriglyceridemia in humans and mice (12, 13). The activity of LPL in different tissues is meticulously regulated to match the mobilization of TG-derived fatty acids to the local tissue demand (7).

In the past decades, many studies have examined potential sex differences in LPL activity. According to Perreault *et al.* (14) (2004), basal levels of muscle and adipose tissue LPL activity were not different between men and women. By contrast, numerous other human studies found that levels of LPL activity in various fat depots, including abdominal, femoral, gluteal, and omental fat, were significantly higher in women than men, as observed in the fasted and/or postprandial state (14, 15, 16, 17, 18, 19, 20). LPL activity seems to be specifically elevated in female adipose tissue and not in female skeletal muscle (19, 20). Collectively, there is convincing evidence that adipose tissue LPL activity is higher in women than in men, which likely explains the lower fasting and postprandial plasma TG levels in women.

Currently, the mechanisms behind the higher adipose LPL activity and resulting lower fasting/postprandial lipemia in women are not fully understood yet. The activity of LPL is regulated by numerous factors, including by three members of the Angiopoietin-like protein family (ANGPTL): ANGPTL3, ANGPTL4, and ANGPTL8 (16, 21). These proteins are expressed in a tissue-specific manner and play a central role in plasma TG partitioning during feeding and fasting by modulating LPL activity (22). ANGPTL3 is exclusively produced in the liver and cooperates with ANGPTL8 in the fed state to inhibit LPL activity in oxidative tissues such as the heart and brown fat (23, 24). ANGPTL8 is primarily produced in the liver and adipose tissue and exerts its effects on LPL activity through interactions with ANGPTL3 and ANGPTL4 (22). ANGPTL4 is produced by multiple tissues and organs, including the liver, adipose tissue, heart, and macrophages, and mainly plays a role in LPL regulation in the fasted state (11). Overexpression of ANGPTL4 and injection of recombinant ANGPTL4 lead to hypertriglyceridemia in mice (25, 26, 27, 28), whereas antibody-mediated inactivation, genetic silencing, and complete genetic ablation of ANGPTL4 significantly decrease plasma TG levels (25, 29, 30, 31). Consistent with the preclinical data, human genetic studies indicate that loss-of-function variants in ANGPTL4 are associated with lower plasma TG levels and also a lower risk of CAD (32, 33, 34). The abundant genetic evidence coupled with a comprehensive understanding of its mechanism of action renders ANGPTL4 a highly attractive pharmacological target for plasma TG lowering.

So far, nearly all preclinical studies on ANGPTL4 have been performed in male mice, with some exceptions (25, 35, 36, 37). As a consequence, little is known about ANGPTL4 action in females. Specifically, it is unclear if the inactivation of ANGPTL4 has similar lipid-lowering effects in females as it does in males. Furthermore, it is unclear if ANGPTL4 may be involved in the sexual dimorphism in plasma TG metabolism and adipose LPL activity. Given that ANGPTL4 is a highly anticipated pharmacological target for improving dyslipidemia and reducing cardiovascular risk, it is important to address these questions. Accordingly, this study aimed to validate the lipid-lowering effect of ANGPTL4 inactivation on plasma TG metabolism in females and examine the potential sexually dimorphic regulation and functional role of ANGPTL4 in governing plasma TG metabolism. We also looked into the potential sexually dimorphic regulation of ANGPTL8 and LPL.

## Materials and methods

### Human samples

Human liver, subcutaneous adipose tissue, and/or plasma samples were obtained from three different studies. The Mondial study is a cross-sectional study of 76 male and female patients undergoing bariatric surgery at the Rijnstate hospital/Vitalys clinics in Arnhem. Subjects were aged 18–60 years, had a BMI >35 kg/m^2^, and met the criteria for surgery from the Interdisciplinary European Guidelines for Surgery for Severe (Morbid) Obesity. A complete description of the study and the details of the sampling can be found elsewhere (38). The study was approved by the local ethics committee of the Rijnstate hospital Arnhem.

The BellyFat study is a parallel controlled-feeding trial in which 110 healthy participants aged 40–70 years with abdominal obesity (BMI > 27 kg/m^2^ or waist circumference > 88 cm for females and > 102 cm for males) were randomly assigned to one of three different energy-restricted diets. In this manuscript, only the subcutaneous adipose samples collected at baseline before the intervention were used (38, 39). The study was approved by the Medical Ethics Committee of Wageningen University and registered at clinicaltrials.gov (NCT02194504).

The MARIS study is a parallel controlled-feeding trial in which 60 healthy participants aged 40–65 years with a BMI >25 kg/m^2^ or a waist circumference ≥94 cm for men and ≥80 cm for women were randomly assigned to three different diets. In this manuscript, only the subcutaneous adipose tissue samples collected at baseline before the intervention were used (40, 41). The study was approved by the Medical Ethics Committee of Wageningen University and registered at clinicaltrials.gov (NCT00405197).

All human studies were conducted in accordance with the Helsinki Declaration of 1975 as revised in 1983.

### Animal studies

#### Study 1

Animal studies were performed in male and female three- to four-month-old wild-type, *Angptl4*-transgenic (Tg), and *Angptl4*−/− mice. All mice are backcrossed on a pure C57Bl/6J background for multiple generations (>10). Wild-type and *Angptl4*-Tg mice are littermates. *Angptl4*−/− mice were obtained via homologous recombination of embryonic stem cells and lack part of the *Angptl4* gene, resulting in a nonfunctional ANGPTL4 protein (42). *Angptl4*-Tg mice overexpress the *Angptl4* gene in all tissues that normally express *Angptl4*, including white and brown adipose tissue, heart, skeletal muscle, and liver. The details of this mouse model have been previously described (28). The *Angptl4*-Tg mice exhibit an amplified response in *Angptl4* expression in response to *Angptl4*-regulating stimuli such as fasting. Male (11 *Angptl4*-Tg, 9 WT, and 13 *Angptl4*−/−) and female (11 *Angptl4*-Tg, 5 WT, and 17 *Angptl4*−/−) mice were housed in group cages under a 12 h light/12 h dark cycle and fed a standard chow diet after weaning. Mice were fasted for 24 h and euthanized between 8.30 and 10.30 a.m. Blood was collected via orbital puncture under isoflurane anesthesia. Immediately thereafter, the mice were euthanized by cervical dislocation, after which tissues were excised and snap-frozen in liquid nitrogen followed by storage at −80°C.

#### Study 2

Male (11 *Angptl4*-Tg, 17 WT, and 15 *Angptl4*−/−) mice were housed in group cages under a 12 h light/12 h dark cycle and fed a standard chow diet after weaning. The mice were euthanized between 8.30 and 11.00 a.m. and were fasted for 24 h or in the ad libitum fed state. The animal studies were approved by the central committee on animal experimentation and the local animal welfare committee of Wageningen University (2007020.c; AVD10400202115283, 2021.W-0016.007).

### Quantification of plasma parameters

Blood samples were collected into EDTA-coated tubes and centrifuged at 4°C for 15 min at 5,000 rpm. Plasma was collected and stored at −80°C. The plasma concentration of various metabolites was determined using specialized kits: triglycerides (Liquicolor, Human GmbH, Wiesbaden, Germany), cholesterol (Diasys, Diagnostics Systems GmbH, Holzheim, Germany), glucose (Diasys), and non-esterified fatty acids (NEFA, Instruchemie, Delfzijl, The Netherlands), following the instructions of the manufacturer. Plasma lipoprotein profiling was performed by HPLC as a lipoprotein analysis service by LipoSEARCH (Tokyo, Japan) using 10 μl of pooled plasma. Plasma ANGPTL4 concentrations were determined using the ELISA kit from R&D systems (Cat: DY3485, R&D systems, the Netherlands) according to the manufacturer's protocol.

### LPL activity assay

LPL activity in adipose tissue of the fasted male and female mice was measured using a previously described method (37). Frozen adipose tissue samples were finely minced using surgical scissors and resuspended in LPL assay buffer (25 mM NH_4_Cl, 5 mM EDTA, 0.01% SDS, 45 U/ml heparin, 0.05% Zwittergent® 3–14 Detergent (Sigma-Aldrich; 693017)) containing protease inhibitor (Roche). The tissue lysate was then vortexed and incubated on ice for 30 min with intermittent disruption with surgical scissors. After centrifugation at 15,000 *g* and 4°C for 15 min, protein concentrations of the supernatant were equalized using a Pierce BCA kit (Thermo Fisher Scientific) before assay activity. Supernatants were combined with the assay buffer (0.6 M NaCl, 80 mM Tris-HCl, pH 8, 6% fatty-acid free BSA, and 1% of the EnzChek lipase fluorescent substrate (Thermo Fisher Scientific; E33955)) in 96-well black clear-bottom plates. Fluorescence was measured in technical duplicates for each lysate (0–30 min, 37°C) on a Spectra Max i2 plate reader (Molecular Devices). Relative lipase activity was determined by calculating the linear slope of the curve and subtraction of background (assay buffer only) slope readings.

### LPL activity assay

LPL activity in adipose tissue of the fed and fasted male mice was quantified using an [^3^H] oleic acid-labeled triolein substrate, as previously described (29).

### Cell culture and treatments

#### Mouse primary hepatocytes

Primary mouse hepatocytes were isolated from WT C57Bl/6J mice, using a two-step collagenase perfusion technique as previously described with a modification that the Liberase stock solution was replaced with the Collagenase solution (0.4 mg/ml, Sigma-Aldrich; 693017) (43). Isolated primary mouse hepatocytes were seeded into collagen-coated 12-well plates at a density of 2 × 10^5^ cells per well and maintained in a humidified chamber at 37°C with 5% CO_2_. After 3 h, the medium was refreshed with warm maintenance medium and left overnight.

The next day, cells were respectively treated for 24 h with Wy-14643 (10 μM), cortisol (550 nM), estradiol (10 nM), testosterone (10 nM), progesterone (10 nM), or rosiglitazone (1 μM). All chemicals were purchased from Sigma-Aldrich and prepared in pure methanol. An equivalent volume of methanol was added to untreated cells as vehicle controls. The concentrations of sex hormones were selected based on the physiologically relevant concentrations observed in human plasma (44).

#### Mouse SVF-derived adipocytes

Inguinal white adipose tissue was dissected from C57Bl/6J mice in an ice-cooled transport medium (DMEM plus 1% fatty acid-free BSA (Sigma-Aldrich)). Tissues of 3–5 mice were pooled, minced with scissors, and digested in the collagenase solution (DMEM, 3.2 mM CaCl_2_, 15 mM Hepes, 0.5% BSA-FA free, 10% FCS, and 1.5 mg/ml collagenase type II (Sigma-Aldrich; C6885)) at 37°C for 30 min. The digested tissue suspensions were then filtered by a 100-μm cell strainer and centrifuged at 1,600 rpm for 10 min at room temperature. The supernatant was removed and the pellet containing the stromal vascular fraction was re-suspended in erythrocyte lysis buffer (155 mM NH_4_Cl, 12 mM NaHCO_3_, 0.1 mM EDTA) and incubated for 2 min at room temperature. After neutralization, cell mixtures were centrifuged at 1,200 rpm for 5 min. The cells were then re-suspended in complete DMEM medium (DMEM plus 10% FCS and 1% penicillin/streptomycin) and seeded into cell culture flasks to around 90% confluency.

Upon confluency, cells were differentiated as previously described with minor modifications (45). Briefly, cells were then resuspended and seeded into the desired culture plates with a density of 15,000 cells/cm^2^ in complete DMEM medium. Two days post-confluence (i.e., day 0), cells were switched to the induction medium (0.5 mM 3-isobutyl-1-methylxanthine (Sigma-Aldrich; I5879), 1 μM dexamethasone (Sigma-Aldrich; D4902), 7 μg/ml insulin (Sigma-Aldrich; I2643), and 1 μM rosiglitazone (Sigma-Aldrich; R2408)). After 3 days, the medium was changed to the insulin medium (complete DMEM medium with 7 μg/ml insulin). Subsequently, the medium was refreshed every 2–3 days. After 10 days of differentiation, cells were switched back to complete DMEM medium for 2–3 days, after which experiments were performed.

Differentiated primary adipocytes were treated with cortisol (550 nM), estradiol (10 nM), testosterone (10 nM), or rosiglitazone (1 μM). After 24 h, cells were washed and harvested for the subsequent gene and protein expression analysis.

#### 3T3-L1 adipocytes

3T3-L1 fibroblasts were amplified in complete DMEM medium (DMEM supplemented with 10% FCS and 1% penicillin/streptomycin) and subsequently seeded into the desired plates (15,000 cells/cm^2^). Upon confluency, cells were differentiated according to the standard protocol for 3T3-L1 cells (42, 45).

Differentiated 3T3-L1 adipocytes were treated with cortisol (550 nM), estradiol (10 nM), testosterone (10 nM), or rosiglitazone (1 μM). After 24 h, cells were washed and harvested for the subsequent gene and protein expression analysis.

### RNA isolations and quantitative PCR

Tissues and cells were homogenized in TRIzol (Invitrogen) by using the Qiagen TissueLyser II and stainless steel beads or by pipetting up and down. Total RNA was isolated using the RNeasy Micro kit from Qiagen (Venlo, The Netherlands). Subsequently, 500 ng of RNA was used to synthesize cDNA using the iScript cDNA synthesis kit (Bio-Rad Laboratories, Veenendaal, The Netherlands). Messenger RNA levels of various genes were determined by reverse transcription-quantitative PCR using SensiMix (Bioline; GC Biotech, Alphen aan den Rijn, The Netherlands) on a CFX384 real-time PCR detection system (Bio-Rad Laboratories, Veenendaal, The Netherlands). Cyclophilin and/or 36b4 were used as housekeeping genes for normalization. Primers were synthesized by Eurogentec (Seraing, Belgium).

RNA isolation and quantitative PCR of liver samples from the Mondial study were carried out as previously described (38).

### Protein isolations and Western blot

Tissues and cells were lysed in RIPA lysis and extraction buffer (Thermo Fisher Scientific) with protease and phosphatase inhibitors (Roche). Following homogenization, lysates were placed on ice for 30 min and centrifuged two or three times at 13,000 *g* for 10 min at 4°C to remove fat and/or cell debris. Protein concentration was determined using a Pierce BCA kit (Thermo Scientific), and equal amounts of protein were diluted with 4× Laemmli sample buffer (Bio-Rad). Protein lysates (10–20 μg of protein per lane) were loaded on an 8–16% gradient Criterion gel (Bio-Rad) and separated by SDS gel electrophoresis. Proteins were transferred to a polyvinylidene difluoride membrane using a Transblot Turbo System (Bio-Rad). Membranes were probed with a rabbit anti-mouse ANGPTL4 antibody (1:2,000, #742, homemade) (46, 47), a rabbit anti-human ANGPTL4 antibody (1:1,000, #1187, homemade) (38, 46, 48), a goat anti-mouse LPL antibody (1:5,000) (49), a mouse anti-mouse ANGPTL8 antibody (1:500) (50), a rabbit anti-human/mouse HSP90 antibody (1:2,000 or 1:5,000, #4874S; Cell Signaling), and a rabbit anti-human/mouse β-Actin antibody (1:2,000, #4970S; Cell Signaling). Secondary antibodies were rabbit anti-mouse (1:5,000, #G21040, ThermoFisher Scientific), rabbit anti-goat (1:5,000; #AP106P, Sigma-Aldrich), and goat anti-rabbit (1:5,000; #AP187P, Sigma-Aldrich). Blocking and incubations with primary and secondary antibodies were done in Tris-buffered saline, pH 7.5, with 0.1% Tween-20 (TBS-T) and 5% (w/v) skimmed milk. In between, membranes were washed in TBS-T. Primary antibodies were applied overnight at 4°C, and secondary antibody was applied for 1 h at room temperature. Blots were visualized using the ChemiDoc MP system (Bio-Rad) and Clarity ECL substrate (Bio-Rad).

Human subcutaneous adipose tissue samples were processed and analyzed by Western blotting as previously described (38). Human liver samples (∼30–40 mg) were processed by adding lysis buffer, followed by homogenization using TissueLyser. Lysates were placed on ice for 30 min and analyzed for protein concentration using a Pierce BCA kit (Thermo Fisher Scientific). Further processing and loading of the samples on an 8–16% gradient Criterion gel (Bio-Rad), separation by SDS gel electrophoresis, transfer, and blotting were carried out as described above.

### Microarray analysis

Baseline adipose tissue samples were available from 31 subjects (16 male, 15 female) from the Maris study and 72 subjects (34 male, 38 female) from the Bellyfat study. The microarray analysis of these adipose tissue samples has been previously described (39, 41).

Liver from fed and 24 h-fasted *Angptl4*-Tg, wild-type, and *Angptl4*−/− mice were analyzed by microarray. Microarray analysis was performed on individual mouse livers of 5 mice per group for a total of 30 arrays. Total RNA from mouse liver was extracted with TRIzol reagent (Invitrogen) and subsequently purified and DNase treated using the SV Total RNA Isolation System (Promega, Leiden, The Netherlands). RNA quality was measured on an Agilent 2100 Bioanalyzer (Agilent Technologies, Amsterdam, The Netherlands) using 6,000 Nano Chips according to the manufacturer's instructions. RNA was judged as suitable for array hybridization only if samples showed intact bands corresponding to the 18S and 28S rRNA subunits, displayed no chromosomal peaks or RNA degradation products, and had an RNA integrity number >8.0. Five micrograms of RNA were used for one cycle cRNA synthesis (Affymetrix, Santa Clara, CA). Hybridization, washing, and scanning of Affymetrix Nutrigenomics Organisation (NuGO) Mouse Arrays were carried out according to standard Affymetrix protocols. The NuGO arrays represent custom-designed Affymetrix GeneChip arrays, designed by the European NuGO and manufactured by Affymetrix. These NuGO microarrays contain in part common probe sets that are also present on the standard Affymetrix arrays and in part newly designed probe sets.

Scans of the Affymetrix arrays were processed using packages from the Bioconductor project (51). Arrays were normalized with quantile normalization, and expression levels of probe sets were calculated using the robust multichip average method (45, 52). *P* values were calculated using an intensity-based moderated T-statistic. Genes were considered to be significantly changed when raw *P* <0.001 (53). Functional analysis of the array data was performed using Gene Set Enrichment Analysis (54).

Two open sources transcriptome datasets were used. mRNA levels of Angiopoietin-like proteins in livers and adipose tissue of female and male subjects were obtained from the Genotype-Tissue Expression (GTEx) project (gtexportal.org) (55). mRNA levels of Angiopoietin-like proteins in livers and adipose of female and male mice were obtained from the GEO dataset GSE23401.

### Statistics

Statistical analysis was carried out in GraphPad using Student’s *t* test, one-way ANOVA followed by Dunnett’s multiple comparisons test, or two-way ANOVA. *P* <0.05 was considered as statistically significant.

## Results

### Sexual dimorphism in ANGPTL3, ANGPTL4, and ANGPTL8 expression in humans

First, we examined potential sexual dimorphism in ANGPTL4 levels in humans. In this analysis, ANGPTL3, ANGPTL8, and LPL were included as well. Information was collected on ANGPTLs and LPL mRNA and protein levels in human liver, adipose tissue, and plasma, if available.

**Fig. 1:**
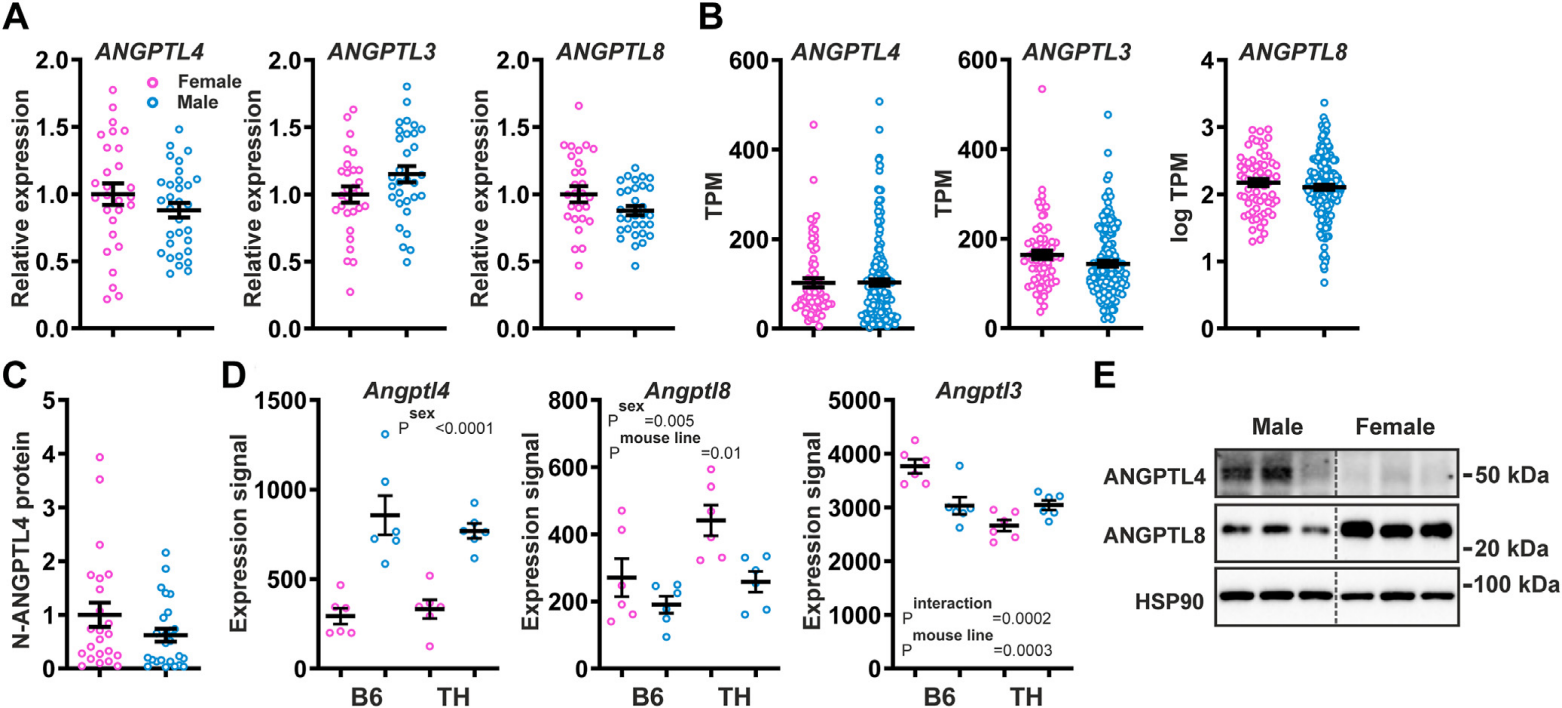
Comparison of hepatic expression of Angiopoietin-like proteins between females and males. A: Relative mRNA levels of Angiopoietin-like proteins in the livers of female and male bariatric surgery patients, as determined by qPCR. The mean expression level in females was set at 1. B: mRNA levels of Angiopoietin-like proteins in the livers of female and male subjects, based on bulk RNA-seq data generated in the Genotype-Tissue Expression (GTEx) project and expressed as transcripts per million (gtexportal.org) (55). C: Relative levels of N-terminal ANGPTL4 protein in the livers of female and male bariatric surgery patients, as determined by Western blot. The mean level in females was set at 1. D: Relative mRNA levels of Angiopoietin-like proteins in the livers of 5-week-old female and male C57BL/6J (B6) and TALLYHO/Jng (TH) mice fed a high-fat diet, based on bulk RNA-seq (GSE23401). E: ANGPTL4 and ANGPTL8 protein levels in the livers of 3–4-month-old female and male C57BL/6J mice, as determined by Western blot. The *gray* dotted line indicates that lanes were not adjacent but pasted from different sections of the gel. Data were analyzed statistically either by unpaired Student's *t* test or two-way ANOVA. Differences/effects were not statistically significant unless specifically indicated. ANGPTL, Angiopoietin-like protein family.

In human liver, no significant differences in *ANGPTL4*, *ANGPTL3*, and *ANGPTL8* mRNA levels were observed between fasted female and male bariatric surgery patients (Fig. 1A). Furthermore, using a large open-source transcript-level dataset (GTEx), no significant differences in liver *ANGPTL4*, *ANGPTL3*, and *ANGPTL8* transcript levels were observed between the two sexes (Fig. 1B). Consistent with the *ANGPTL4* mRNA data, liver ANGPTL4 protein levels were not significantly different between the female and male bariatric surgery patients (Fig. 1C and supplemental Fig. S1). In mouse liver, using an open source dataset derived from two mouse strains, *Angptl4* mRNA levels were higher in males than females in young mice, but not in adult mice (Fig. 1D and supplemental Fig. S2). In contrast, *Angptl8* mRNA levels were higher in females than males in adult mice, but less so in young mice (Fig. 1D and supplemental Fig. S2). *Angptl3* mRNA levels were not consistently different between females and males in young and adult mice (Fig. 1D and supplemental Fig. S2). Mirroring the mRNA levels, ANGPTL4 protein levels were higher in male mice, whereas ANGPTL8 protein levels were higher in female mice (Fig. 1E).

**Fig. 2:**
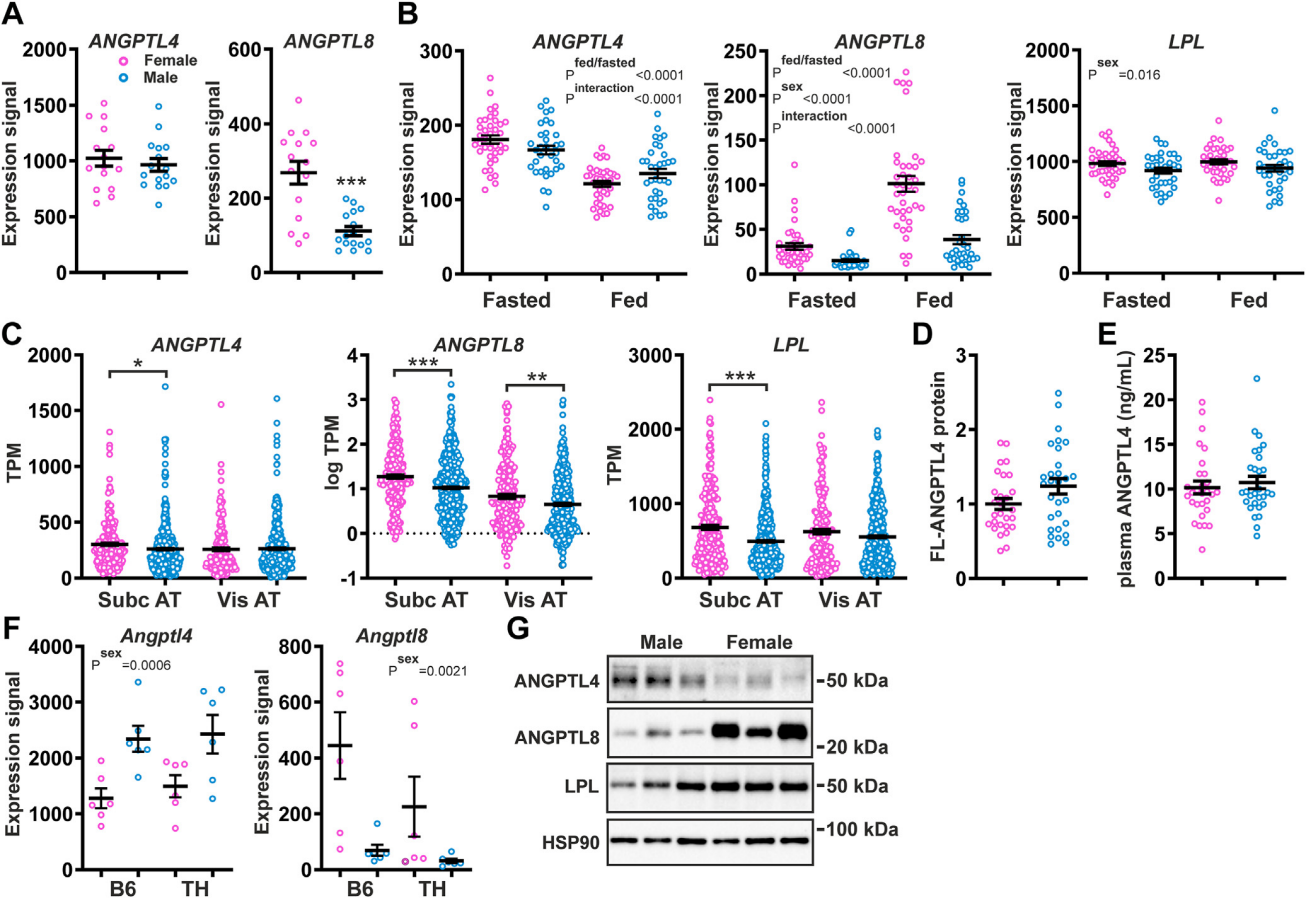
Comparison of adipose tissue expression of Angiopoietin-like proteins and lipoprotein lipase between females and males. A: Relative mRNA levels of *ANGPTL4* and *ANGPTL8* in the adipose tissue of female and male abdominally overweight subjects in the fasted state, as determined by microarray analysis. B: mRNA levels of *ANGPTL4*, *ANGPTL8,* and *LPL* in the adipose tissue of female and male abdominally overweight subjects in the fasted and postprandial state, as determined by microarray analysis. C: mRNA levels of *ANGPTL4*, *ANGPTL8,* and *LPL* in the adipose tissue of female and male subjects, based on bulk RNA-seq data generated in the Genotype-Tissue Expression (GTEx) project and expressed as transcripts per million (gtexportal.org) (55). D: Relative levels of full-length ANGPTL4 protein in adipose tissue of female and male bariatric surgery patients, as determined by Western blot. The mean level in females was set at 1. E: Plasma levels of ANGPTL4 in female and male bariatric surgery patients, as determined by ELISA. F: Relative mRNA levels of *Angptl4* and *Angptl8* in the adipose tissue of 20-week-old female and male C57BL/6J (B6) and TALLYHO/Jng (TH) mice fed a high-fat diet, based on bulk RNA-seq (GSE23401). G: ANGPTL4, ANGPTL8, and LPL protein levels in gonadal adipose tissue of 3–4-month-old female and male C57BL/6J mice, as determined by Western blot. Data were analyzed statistically either by unpaired Student's *t* test or two-way ANOVA. Differences/effects were not statistically significant unless specifically indicated. ANGPTL, Angiopoietin-like protein family.

In human subcutaneous adipose tissue, no significant differences in *ANGPTL4* mRNA levels were observed between fasted female and male abdominally overweight subjects (Fig. 2A). By contrast, adipose *ANGPTL8* mRNA levels were significantly higher in the female than male subjects (Fig. 2A). These findings were confirmed in another study in abdominally overweight subjects in both the fasted and post-prandial state (Fig. 2B). In this study, adipose *LPL* mRNA levels were significantly higher in females than in males. Analysis of open-source data from the GTEx project confirmed the significantly higher *ANGPTL8* mRNA levels in subcutaneous and visceral adipose tissue of females compared to males (Fig. 2C). *ANGPTL4* mRNA in subcutaneous adipose tissue was slightly but significantly lower in males than in females, whereas *ANGPTL4* mRNA in visceral adipose tissue was not different between the two sexes (Fig. 2C). *LPL* mRNA was significantly higher in female than male subcutaneous adipose tissue and was not significantly different between female and male visceral adipose tissue (Fig. 2C). In line with the *ANGPTL4* mRNA data, levels of full-length ANGPTL4 protein in subcutaneous adipose tissue, as determined in the bariatric surgery patients, were not different between the two sexes (Fig. 2D and supplemental Fig. S3) nor were ANGPTL4 protein levels in plasma (Fig. 2E). In mice, using the above-mentioned open-source dataset, *Angptl8* mRNA levels were higher in females than males, regardless of the mouse strain, diet, and age, while *Angptl4* mRNA levels were higher in male than female 20-week-old mice fed a high-fat diet (Fig. 2F and supplemental Fig. S2). Adipose *Lpl* mRNA levels were not very variable between the sexes (supplemental Fig. S2). Consistent with the mRNA data, ANGPTL8 protein levels were higher in the adipose tissue of female mice than that of male mice, whereas ANGPTL4 protein showed an opposite trend (Fig. 2G).

Elaborating on the plasma ANGPTL4 data, analysis of open-source plasma protein data from the Icelandic cohort using the Somalogic platform indicated that plasma ANGPTL4 levels were slightly higher in males than females (beta = 0.02061485, *P* = 1.19E-10), whereas plasma ANGPTL8 levels were not affected by sex (beta = −0.00603334, *P* = 5.63E-02) (56). By contrast, plasma ANGPTL3 levels were significantly lower in males than in females (beta = −0.1212792, *P* = 2.43E-247).

Taken together, ANGPTL4 mRNA and protein levels in the liver and adipose tissue were higher in male than female mice. By contrast, no sexual dimorphism in ANGPTL4 expression was observed in humans. Pronounced sexual dimorphism was observed for *ANGPTL8* mRNA in humans and ANGPTL8 mRNA and protein in mice, especially in adipose tissue, showing higher levels in females than in males.

### Examination of sexual dimorphism in ANGPTL4 function

**Fig. 3:**
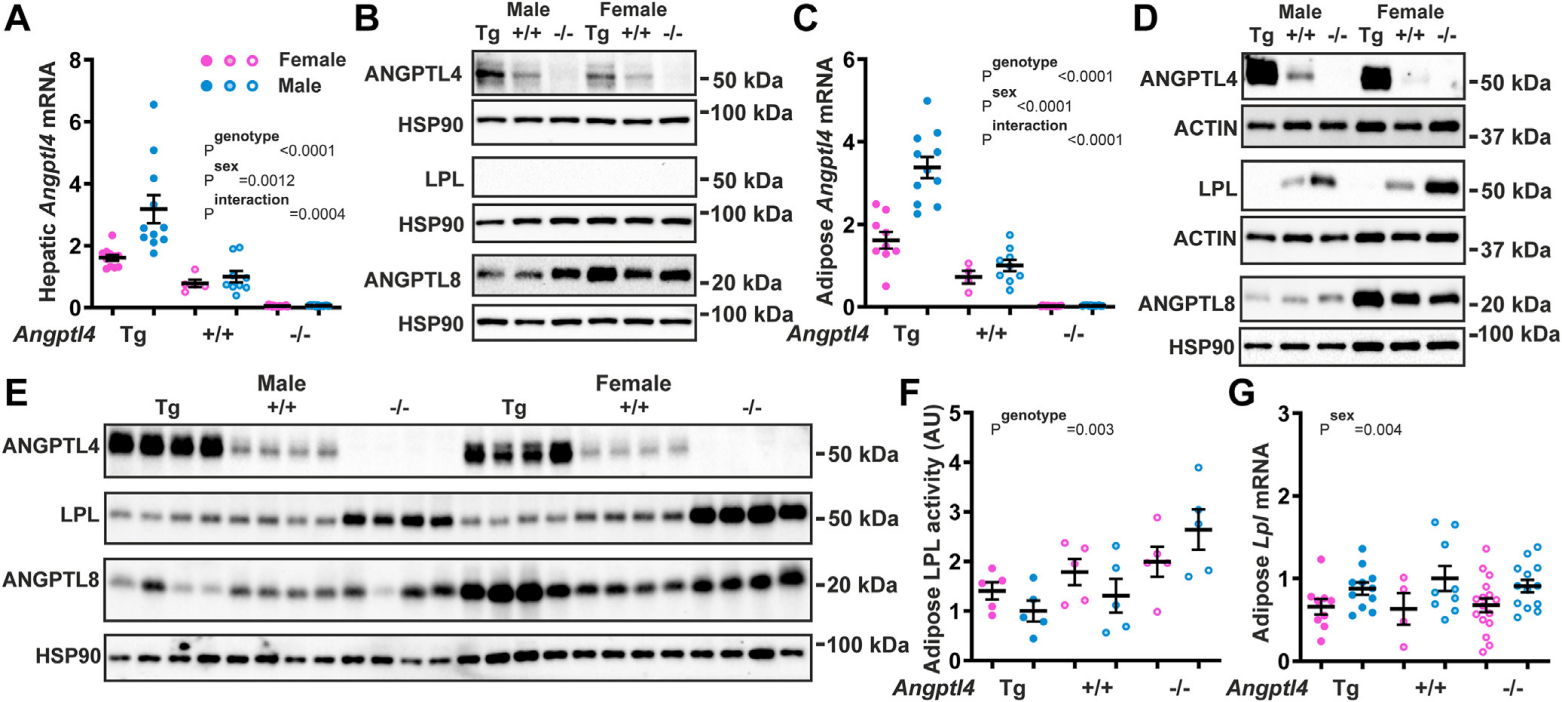
ANGPTL4 mRNA and protein levels in female and male *Angptl4*-Tg, wild-type, and *Angptl4*−/− mice fasted for 24 h. A: Hepatic *Angptl4* mRNA levels in female and male *Angptl4*-Tg, wild-type, and *Angptl4*−/− mice, as determined by qPCR. B: Pooled hepatic ANGPTL4, ANGPTL8, and LPL protein levels in female and male *Angptl4*-Tg, wild-type, and *Angptl4*−/− mice, as determined by Western blot. C: Adipose *Angptl4* mRNA levels in female and male *Angptl4*-Tg, wild-type, and *Angptl4*−/− mice, as determined by qPCR. D: Pooled adipose ANGPTL4, ANGPTL8, and LPL protein levels in female and male *Angptl4*-Tg, wild-type, and *Angptl4*−/− mice, as determined by Western blot. E: Adipose ANGPTL4, ANGPTL8, and LPL protein levels in female and male *Angptl4*-Tg, wild-type, and *Angptl4*−/− mice, as determined by Western blot. F: Adipose LPL activity in female and male *Angptl4*-Tg, wild-type, and *Angptl4*−/− mice, as determined by enzymatic analysis. G: Adipose *Angptl4* mRNA levels in female and male *Angptl4*-Tg, wild-type, and *Angptl4*−/− mice, as determined by qPCR. Quantitative data were analyzed statistically by two-way ANOVA. Differences/effects were not statistically significant unless specifically indicated. ANGPTL, Angiopoietin-like protein family.

To investigate whether the functional effects of ANGPTL4 may differ between the two sexes, we studied male and female whole-body *Angptl4*-transgenic (Tg) mice, wild-type mice, and *Angptl4*−/− mice. *Angptl4* mRNA levels in the liver were consistent with the genotype and significantly higher in the male mice than the female mice, most prominently in the *Angptl4*-Tg mice (Fig. 3A). Similarly, hepatic ANGPTL4 protein levels reflected the *Angptl4* genotype and were higher in male than female mice (Fig. 3B). ANGPTL8 protein levels in the liver were higher in female than male mice, again most prominently in the *Angptl4*-Tg mice (Fig. 3B).

As observed in the liver, *Angptl4* mRNA and ANGPTL4 protein levels in the adipose tissue reflected the *Angptl4* genotype and were significantly higher in male than female mice (Fig. 3C, D). In line with previous studies (47, 57), adipose LPL protein levels were inversely correlated with ANGPTL4 levels (Fig. 3D, E and supplemental Fig. S4). Adipose LPL protein levels were higher in female compared to male *Angptl4*−/− mice, which was abolished by ANGPTL4 (over) expression (Fig. 3D, E). Adipose LPL activity was significantly influenced by the *Angptl4* genotype—showing an inverse relationship with ANGPTL4 levels—but did not exhibit a consistent effect of sex (Fig. 3F). In contrast, adipose *Lpl* mRNA was significantly influenced by sex, as indicated by higher levels in males, but did not show an effect of genotype (Fig. 3G). Adipose ANGPTL8 protein levels were generally higher in female than male mice (Fig. 3D, E). As observed in the liver, the highest ANGPTL8 protein levels in the adipose tissue were observed in *Angptl4*-Tg mice. In summary, hepatic and adipose ANGPTL4 levels were higher in male than female mice, regardless of *Angptl4* genotype, whereas hepatic and adipose ANGPTL8 levels were higher in female than male mice. Interestingly, ANGPTL4 expression abolished the elevated adipose LPL protein content in female compared to male *Angptl4*−/− mice.

**Fig. 4:**
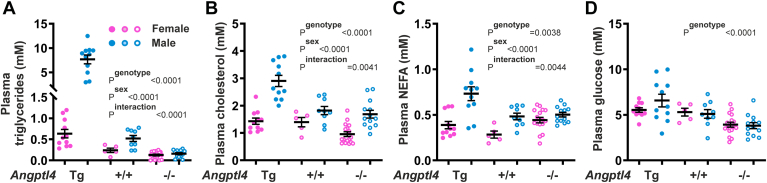
Plasma lipid and glucose levels in female and male *Angptl4*-Tg, wild-type, and *Angptl4*−/− mice fasted for 24 h. Plasma triglycerides (A), cholesterol (B), non-esterified fatty acids (NEFA) (C), and glucose (D) in female and male *Angptl4*-Tg, wild-type, and *Angptl4*−/− mice. Data were analyzed statistically by two-way ANOVA. Differences/effects were not statistically significant unless specifically indicated. ANGPTL, Angiopoietin-like protein family; NEFA, non-esterified fatty acid.

To address whether the effect of ANGPTL4 overexpression and ablation on lipid metabolism may be different between males and females, several plasma lipid parameters were determined. Plasma TG (Fig. 4A), cholesterol (Fig. 4B), and NEFA (Fig. 4C) levels were significantly higher in the male mice than female mice, most prominently in the *Angptl4*-Tg mice. Plasma TG, cholesterol, and NEFA levels were also significantly affected by the *Angptl4* genotype, generally showing a positive correlation with *Angptl4* expression (Fig. 4A–C). For all three parameters, a statistically significant interaction was observed between *Angptl4* genotype and sex. Specifically, the impact of *Angptl4* overexpression and ablation on plasma TG was more pronounced in male mice. Concerning plasma cholesterol, in the male mice, levels were higher in the *Angptl4*-Tg mice than wild-type and *Angptl4*−/− mice, while in the female mice, levels were higher in the *Angptl4*-Tg and wild-type mice than *Angptl4*−/− mice (Fig. 4B). Plasma NEFA showed an interesting pattern, whereby in the male mice, the highest levels were observed in the *Angptl4*-Tg mice, whereas in the female mice, the highest NEFA levels were observed in the *Angptl4*−/− mice (Fig. 4C). Lastly, plasma glucose levels were significantly influenced by the *Angptl4* genotype—showing a downward trend with decreasing *Angptl4* expression—but were unaffected by sex nor showed any interaction between sex and genotype (Fig. 4D). Taken together, sexual dimorphism was observed for the magnitude of the stimulatory effect of ANGPTL4 on plasma TG and cholesterol, whereas for plasma NEFA, sexual dimorphism was observed for the pattern of the effect of ANGPTL4 across genotypes. No sexual dimorphism was observed for the stimulatory effect of ANGPTL4 on plasma glucose.

### Effects of sex hormones on ANGPTL4 expression

Liver and adipose tissue have the highest ANGPTL4 expression level and for a large part account for the functional effects of ANGPTL4 on plasma lipid metabolism (36, 37, 58, 59). Accordingly, we determined whether ANGPTL4 expression in hepatocytes and adipocytes is under the control of sex hormones. Specifically, primary mouse hepatocytes, mouse SVF-derived adipocytes, and 3T3-L1 adipocytes were treated with estradiol or testosterone, followed by the measurement of ANGPTL4 and LPL levels. We also measured *Angptl8* mRNA but unfortunately were unable to capture ANGPTL8 protein in cultured hepatocytes and adipocytes with the available antibody.

**Fig. 5:**
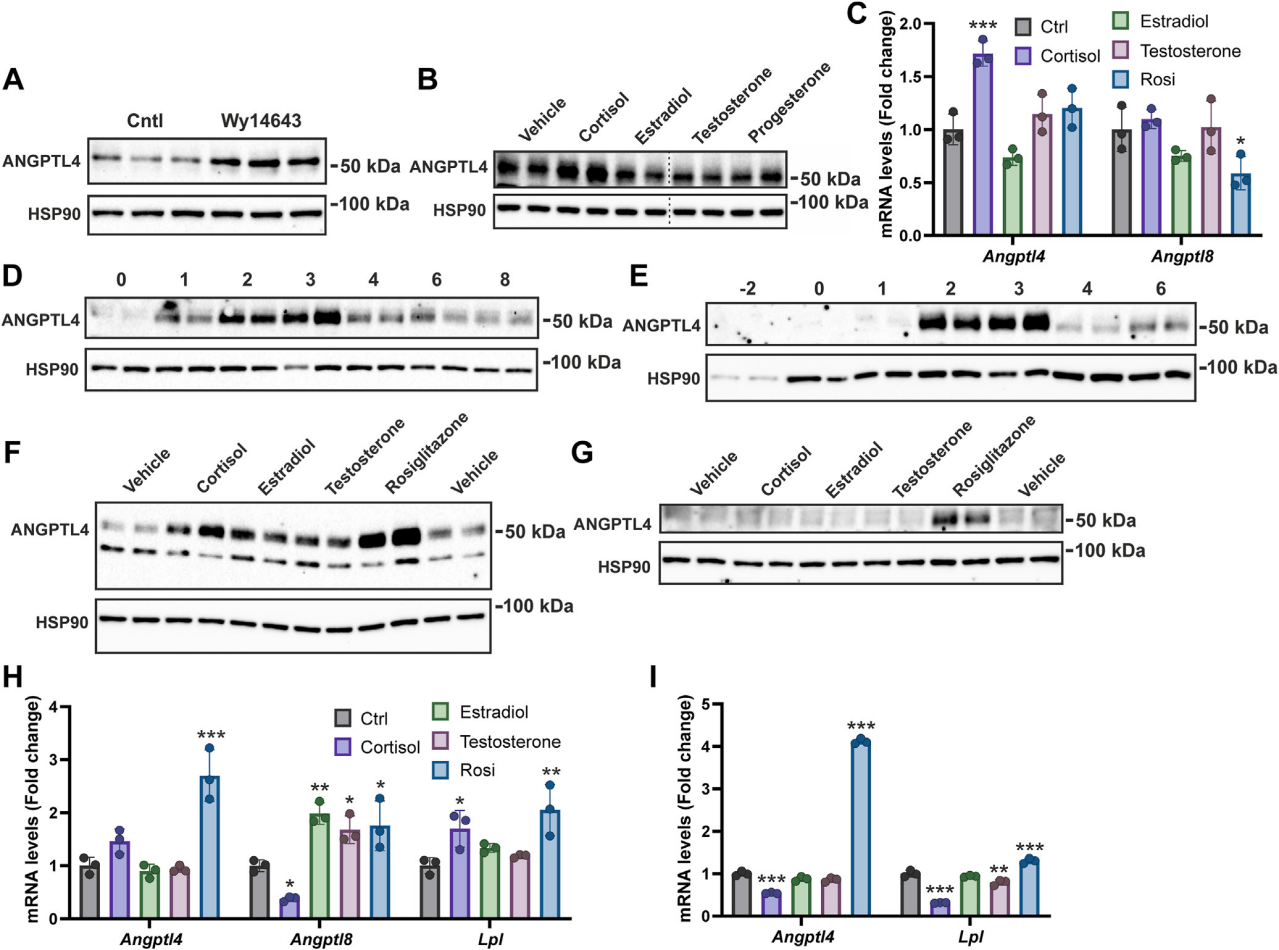
Effects of sex hormones on ANGPTL4 expression in mouse primary hepatocytes, primary SVF-derived adipocytes, and 3T3-L1 adipocytes. A: ANGPTL4 protein levels in primary hepatocytes treated with 10 μM PPARα agonist Wy-14643 for 24 h. B: ANGPTL4 protein levels in primary hepatocytes treated with 550 nM cortisol, 10 nM estradiol, 10 nM testosterone, and 10 nM progesterone for 24 h. The *gray* dotted line indicates that lanes were not adjacent but pasted from different sections of the gel. C: *Angptl4* and *Angptl8* mRNA levels in primary hepatocytes treated with cortisol, estradiol, testosterone, and rosiglitazone. D: ANGPTL4 protein levels in SVF-derived adipocytes during differentiation. E: ANGPTL4 protein levels in 3T3-L1 adipocytes during differentiation. F: ANGPTL4 protein levels in SVF-derived adipocytes treated with cortisol, estradiol, testosterone, and rosiglitazone. G: ANGPTL4 protein levels in 3T3-L1 adipocytes treated with cortisol, estradiol, testosterone, and rosiglitazone. H: *Angptl4*, *Angptl8*, and *Lpl* mRNA levels in SVF-derived adipocytes treated with cortisol, estradiol, testosterone, and rosiglitazone. I: *Angptl4* and *Lpl* mRNA levels in 3T3-L1 adipocytes treated with cortisol, estradiol, testosterone, and rosiglitazone. Data were analyzed statistically by one-way ANOVA followed by Dunnett’s multiple comparisons test. The asterisk indicates statistical significance in comparison to control. ∗*P* < 0.05; ∗∗*P* < 0.01; ∗∗∗*P* < 0.001. ANGPTL, Angiopoietin-like protein family.

ANGPTL4 protein was detectable in primary mouse hepatocytes and was significantly elevated by the PPARα agonist Wy-14643, indicating that primary hepatocytes are suitable for studying ANGPTL4 regulation (Fig. 5A). Cortisol treatment, serving as a positive control (60), upregulated ANGPTL4 mRNA and protein levels by about two-fold. By contrast, estradiol and testosterone did not influence ANGPTL4 mRNA and protein levels nor did they influence *Angptl8* mRNA (Fig. 5B, C). In adipocytes, we first determined the pattern of ANGPTL4 protein during differentiation. Levels of ANGPTL4 protein exhibited a similar trend during differentiation of SVF-derived and 3T3-L1 adipocytes, with ANGPTL4 levels increasing after starting differentiation, peaking on the third day, and subsequently decreasing and stabilizing until the completion of differentiation (Fig. 5D, E). The positive control rosiglitazone significantly increased ANGPTL4 mRNA and protein levels in differentiated SVF-derived and 3T3-L1 adipocytes (Fig. 5F, G), while cortisol treatment significantly increased ANGPTL4 mRNA and protein levels in SVF-derived adipocytes but not in 3T3-L1 adipocytes (Fig. 5F, G). As for the sex hormones, estradiol and testosterone similarly upregulated ANGPTL4 protein in SVF-derived adipocytes (Fig. 5F), whereas these hormones did not alter ANGPTL4 protein levels in 3T3-L1 adipocytes (Fig. 5G). Estradiol and testosterone did not alter *Angptl4* mRNA in either type of adipocyte (Fig. 5H, I). Interestingly, in SVF-derived adipocytes, *Angptl8* mRNA was suppressed by cortisol and induced by estradiol, testosterone, and rosiglitazone (Fig. 5H). Collectively, our results indicate that ANGPTL4 is similarly induced by estradiol and testosterone in SVF-derived adipocytes but is not regulated by these sex hormones in 3T3-L1 adipocytes and primary hepatocytes.

Finally, we examined whether genetic inactivation of ANGPTL4 in humans similarly influences plasma TG in women and men using data form the Global Lipid Genetics Consortium. To that end, we assessed the impact of the ANGPTL4 E40K loss-of-function variant in women and men. GWAS analysis indicated that the effect size of the E40K variant on plasma TG was highly similar in women (−0.235696 s.d. per allele, *P* = 5.91e-236) and men (−0.247202 s.d. per allele, *P* = 7.62e-327) (Table 1), indicating that ANGPTL4 loss of function similarly impacts plasma TG in the two sexes.

**Table 1:** Association of the E40K genotype (rs116843064) with plasma TG in males and females

Sex	N	A	B	C	SE	*P* Value
Female	566,056	147	0.0197	−0.235696	0.00718588	5.91E-236
Male	682,520	142	0.02	−0.247202	0.00639539	7.62E-327

A, number of studies.

B, Pooled Alternative Allele Frequency.

C, Alternative Allele Effect Size.

### Investigation into the role of ANGPTL4 in the liver

**Fig. 6:**
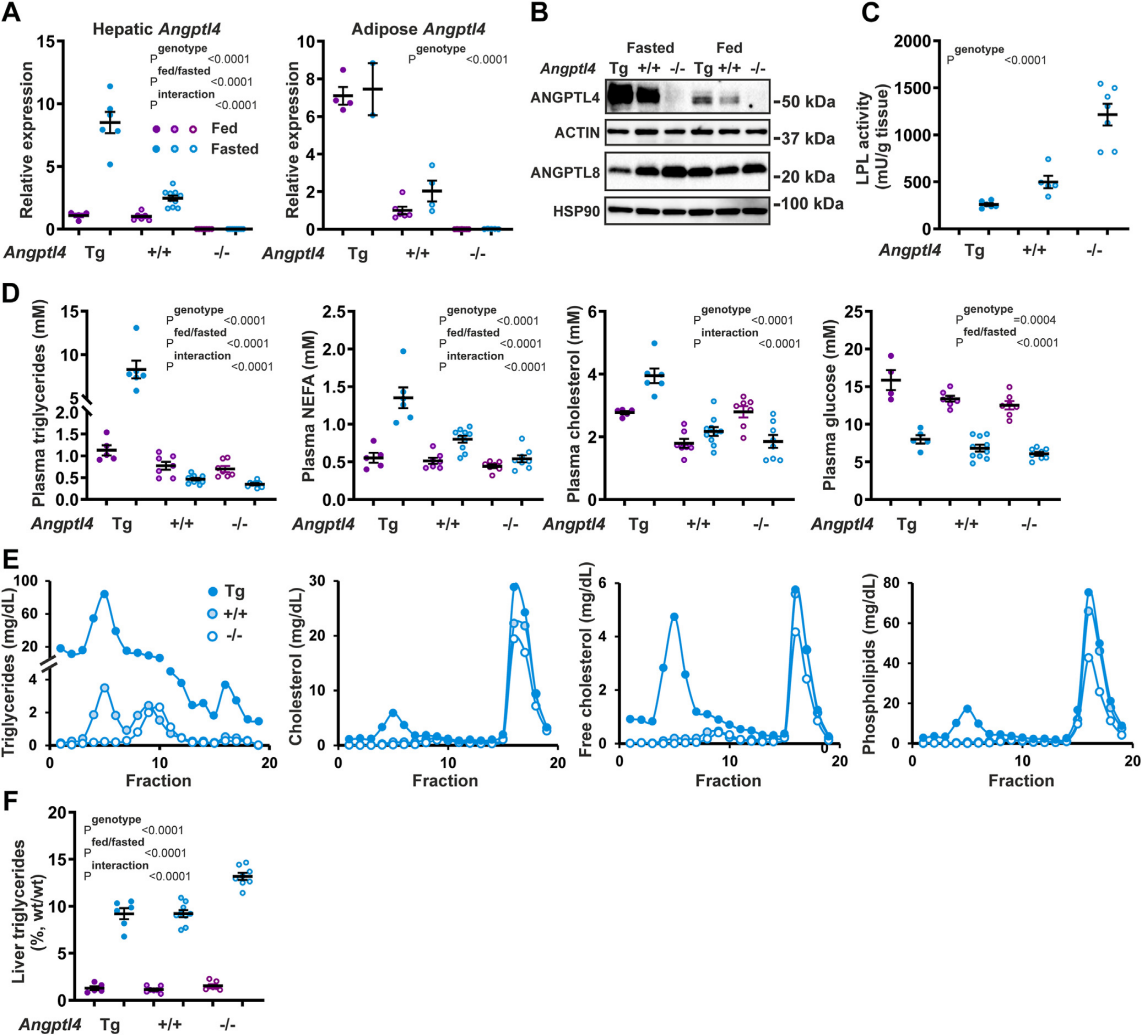
Metabolic phenotype of fed and 24 h-fasted male *Angptl4*-Tg, wild-type, and *Angptl4*−/− mice. A: Hepatic (left panel) and adipose (right panel) *Angptl4* mRNA levels in fed and 24 h-fasted male *Angptl4*-Tg, wild-type, and *Angptl4*−/− mice, as determined by qPCR. B: ANGPTL4 protein levels in the livers of fed and 24 h-fasted male *Angptl4*-Tg, wild-type, and *Angptl4*−/− mice, as determined by Western blot. C: LPL activity in the adipose tissue of 24 h-fasted male *Angptl4*-Tg, wild-type, and *Angptl4*−/− mice. D: Plasma triglycerides, NEFA, cholesterol, and glucose levels in fed and 24 h-fasted male *Angptl4*-Tg, wild-type, and *Angptl4*−/− mice. E: HPLC-based lipoprotein profiling of pooled plasma of 24 h-fasted male *Angptl4*-Tg, wild-type, and *Angptl4*−/− mice. Fractions were analyzed for triglycerides, total cholesterol, free cholesterol, and phospholipids. F: Triglyceride levels in the livers of fed and 24 h-fasted male *Angptl4*-Tg, wild-type, and *Angptl4*−/− mice. ANGPTL, Angiopoietin-like protein family; NEFA, non-esterified fatty acid.

In the next set of experiments, we aimed to further explore the function of ANGPTL4 in the liver during fasting. Considering that the role of ANGPTL4 in the regulation of plasma lipids appears to be consistent between females and males, studies were performed in male *Angptl4*-Tg, wild-type, and *Angptl4*−/− mice only, which were either fasted for 24 h or in the ad libitum fed state. We first confirmed the well-established upregulation of hepatic and adipose *Angptl4* mRNA by fasting (Fig. 6A). Hepatic and adipose *Angptl4* mRNA levels also reflected the genotype. Analysis of hepatic ANGPTL4 protein validated the marked induction by fasting and the effect of genotype (Fig. 6B). Interestingly, in the fasted state, ANGPTL4 protein levels were inversely proportional to ANGPTL8 protein levels (Fig. 6B). No LPL protein could be detected in liver tissue, regardless of nutritional status and genotype (supplemental Fig. S5). As expected, adipose tissue LPL activity, which was only measured in the fasted state, declined with increasing ANGPTL4 levels (Fig. 6C). In line with previous studies (29, 35, 61) and reflecting the ANGPTL4 protein levels, the effect of the *Angptl4* genotype on plasma TG and NEFA levels was much more pronounced in the fasted state than in the fed state (Fig. 6D). Plasma cholesterol showed an interesting pattern; in the fed state, both *Angptl4*-Tg and *Angptl4*−/− mice exhibited increased plasma cholesterol compared to wild-type mice, whereas in the fasted state, plasma cholesterol declined with decreasing ANGPTL4 levels. Plasma glucose was decreased by fasting and declined with decreasing ANGPTL4 levels (Fig. 6D). Overall, the above data verify the stimulatory effect of ANGPTL4 on plasma TG, NEFA, and glucose levels and corroborate the prominent role of ANGPTL4 in metabolic regulation during fasting.

To further explore the influence of ANGPTL4 on cholesterol metabolism, we performed lipoprotein profiling by HPLC on the plasma from the 24 h-fasted mice (Fig. 6E). The TG content of all lipoprotein classes reflected the total plasma TG concentration and was markedly elevated in the *Angptl4*-Tg mice and reduced in the *Angptl4*−/− mice as compared to the wild-type mice. Notably, the amount of cholesterol and phospholipids contained in HDL was also elevated in the *Angptl4*-Tg mice and reduced in the *Angptl4*−/− mice. The amount of unesterified cholesterol in HDL was reduced in the *Angptl4*−/− mice yet unaffected in the *Angptl4*-Tg mice (Fig. 6E).

**Fig. 7:**
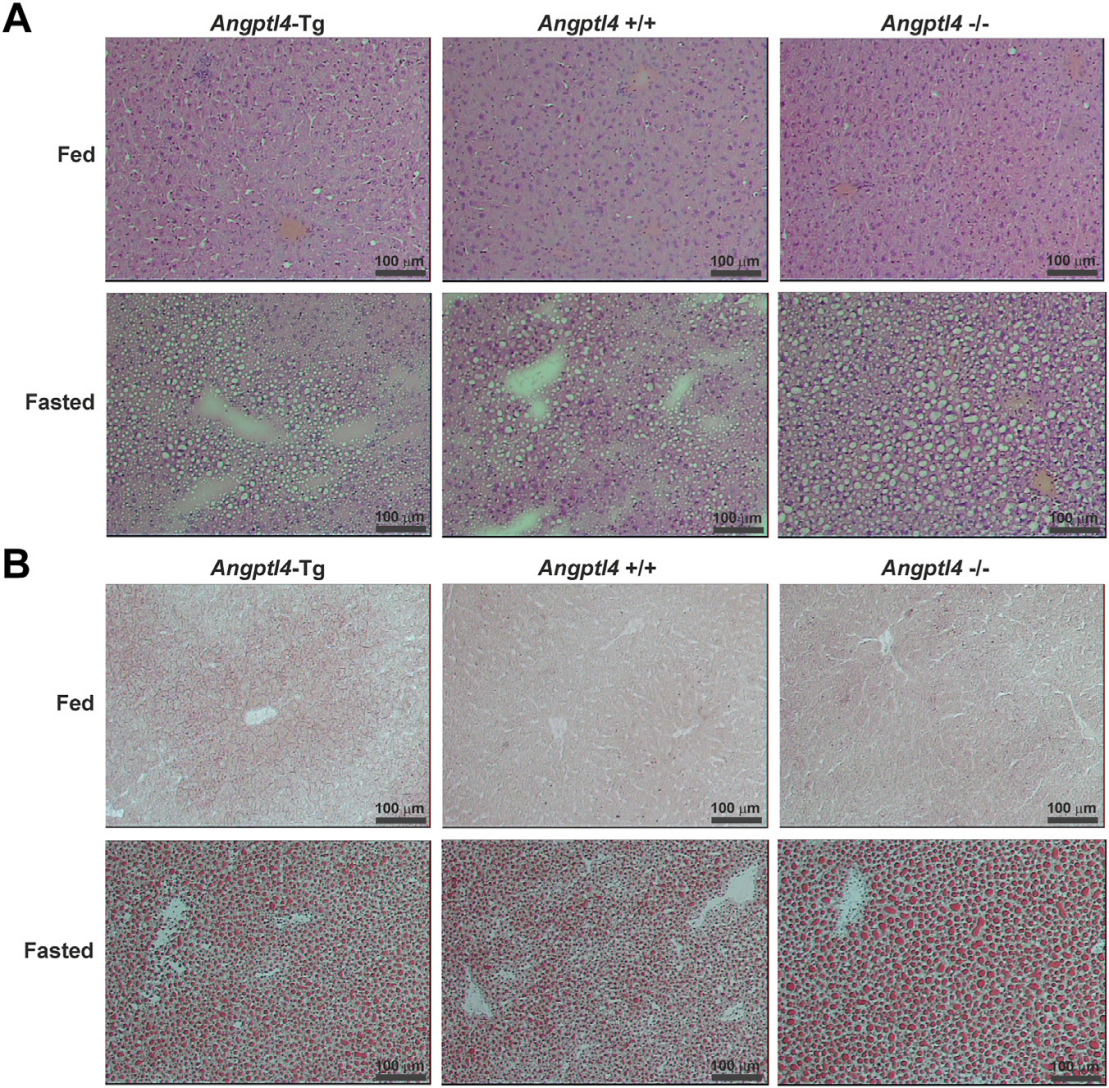
Histology of the livers of fed and 24 h-fasted male *Angptl4*-Tg, wild-type, and *Angptl4*−/− mice. A: Representative H&E staining of livers of fed and 24 h-fasted male *Angptl4*-Tg, wild-type, and *Angptl4*−/− mice. B: Representative Oil Red O staining of livers of fed and 24 h-fasted male *Angptl4*-Tg, wild-type, and *Angptl4*−/− mice. ANGPTL, Angiopoietin-like protein family.

**Fig. 8:**
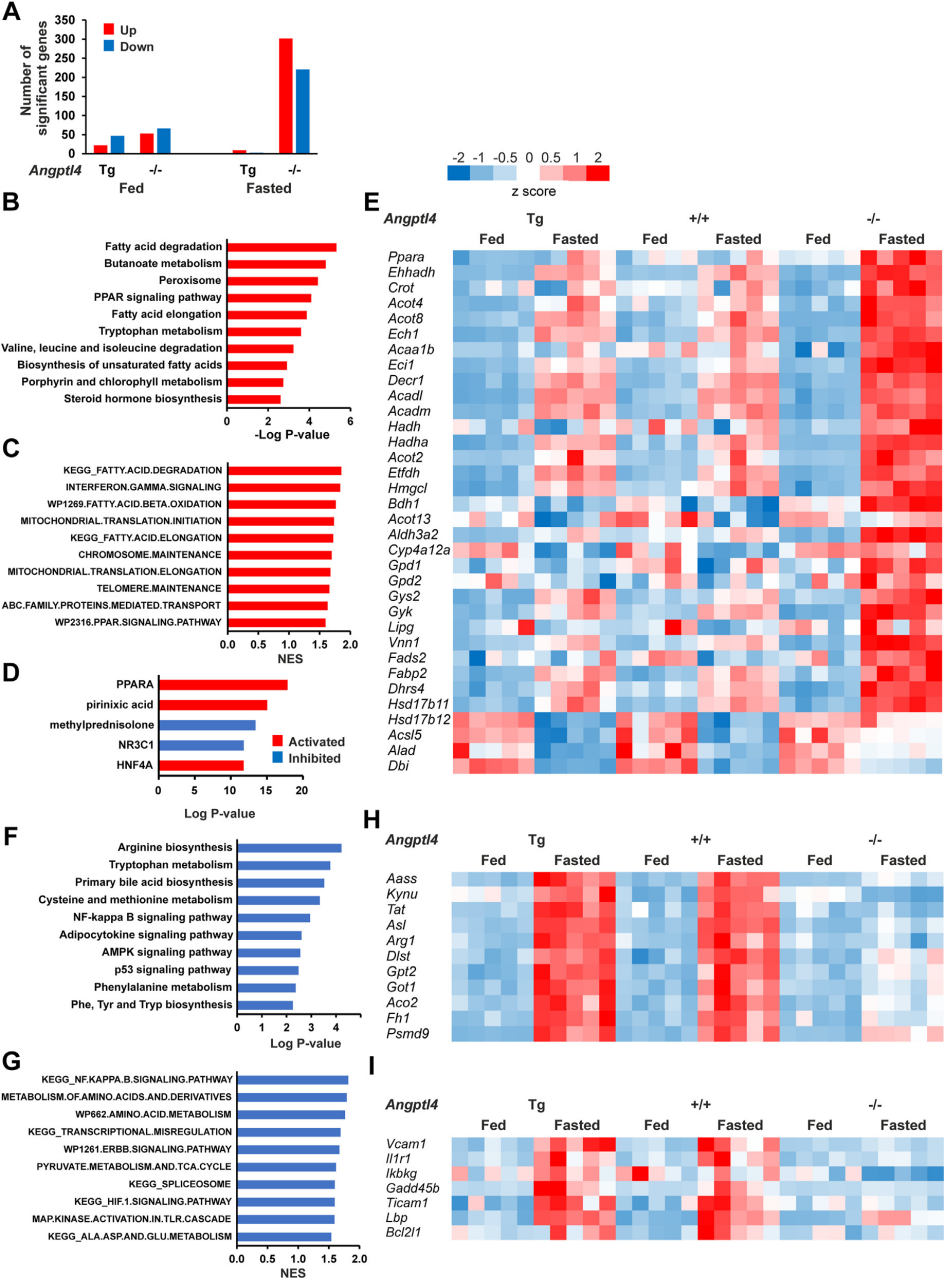
Comparative transcriptome analysis of the livers of fed and 24 h-fasted male *Angptl4*-Tg, wild-type, and *Angptl4*−/− mice. A: Number of differentially expressed genes (IBMT *P* < 0.001) in *Angptl4*-Tg and *Angptl4*−/− mice compared to wild-type mice. B: ENRICHR analysis of the genes significantly induced (FDR *P* < 0.05) in fasted *Angptl4*−/− compared to fasted wild-type mice. The 10 most significant KEGG pathways are shown. C: GSEA showing the 10 most significantly induced gene sets in fasted *Angptl4*−/− compared to fasted wild-type mice ranked according to normalized enrichment score (NES). D: Upstream regulator analysis within Ingenuity Pathway analysis for the comparison between fasted *Angptl4*−/− and fasted wild-type mice. E: Heatmap showing the more pronounced induction by fasting of PPARα and its target genes involved in fatty acid metabolism in *Angptl4*−/− mice compared to wild-type and *Angptl4*-Tg mice. Listed are genes that are significantly induced in fasted *Angptl4*−/− compared to fasted wild-type mice (FDR *P* < 0.05) and are part of the list of established PPARα targets (https://en.wikipedia.org/wiki/Peroxisome_proliferator-activated_receptor_alpha). F: ENRICHR analysis of the genes significantly repressed (FDR *P* < 0.05) in fasted *Angptl*4−/− compared to fasted wild-type mice. The 10 most significant KEGG pathways are shown. G: GSEA showing the 10 most significantly repressed gene sets in fasted *Angptl4*−/− compared to fasted wild-type mice ranked according to normalized enrichment score (NES). H: Heatmap showing the lack of induction of genes involved in amino acid metabolism by fasting in *Angptl4*−/− mice. I: Heatmap showing the lack of induction of genes part of the KEGG NF-κB signaling pathway by fasting in *Angptl4*−/− mice. ANGPTL, Angiopoietin-like protein family; GSEA, Gene Set Enrichment Analysis; IBMT, intensity-based moderated T-statistic.

Further zooming in on the liver, hepatic TG content was significantly increased by fasting, which was enhanced in the *Angptl4*−/− mice (Fig. 6F). The increased lipid storage upon fasting and the enhancement in the *Angptl4*−/− mice were corroborated by H&E staining (Fig. 7A) and Oil Red O neutral lipid staining (Fig. 7B). To further investigate the role of ANGPTL4 in the liver, we performed transcriptome analysis. In comparison to the wildtype, the largest number of differentially expressed genes was observed for the *Angptl4*−/− mice in the fasted state, indicating that ANGPTL4 ablation, but not ANGPTL4 overexpression, has a more pronounced effect on hepatic gene expression during fasting than during feeding (Fig. 8A).

Given that the role of ANGPTL4 in lipid metabolism is mainly evident during fasting, we performed pathway analysis to identify pathways/gene sets altered by ANGPTL4 ablation in the fasting state, thus comparing fasted wild-type and *Angptl4*−/− livers. According to ENRICHR (Fig. 8B) and Gene Set Enrichment Analysis (Fig. 8C), ANGPTL4 ablation was associated with upregulation of several gene sets related to intracellular fatty acid metabolism, including fatty acid oxidation, fatty acid elongation, and PPAR signaling. In agreement with the activation of PPARα, upstream regulator analysis within Ingenuity Pathway analysis yielded PPARα as the most significant upstream regulator (Fig. 8D). A heatmap illustrates the more pronounced fasting-mediated induction of PPARα and its target genes involved in fatty acid metabolism in *Angptl4*−/− mice compared to wild-type and *Angptl4*-Tg mice (Fig. 8E). By contrast, ANGPTL4 ablation was associated with the downregulation of several gene sets related to amino acid metabolism and inflammation (Fig. 8F, G), which are biological processes that are suppressed by PPARα (62, 63, 64). As illustrated by the heatmap, the induction by fasting of genes involved in amino acid metabolism is slightly enhanced in the *Angptl4*-Tg mice and almost completely abolished in the *Angptl4*−/− mice (Fig. 8H). Similarly, the induction by fasting of genes involved in the NF-kB signaling was abolished in the *Angptl4*−/− mice (Fig. 8I). Collectively, these data suggest that ANGPTL4 ablation leads to the activation of PPARα in the liver.

## Discussion

ANGPTL4 is a highly anticipated pharmacological target for improving dyslipidemia and reducing cardiovascular risk. Evidence abounds indicating that ANGPTL4 impacts plasma TG levels via LPL inhibition (11). So far, most pre-clinical studies on ANGPTL4 have been performed in male mice. Here, we aimed to examine the potential sexually dimorphic regulation and functional role of ANGPTL4 in governing plasma TG metabolism and validate the lipid-lowering effect of ANGPTL4 inactivation on plasma TG metabolism in females. The following key findings were made:

*1*) ANGPTL4 mRNA and protein levels in the liver and adipose tissue were higher in male than female mice. By contrast, no sexual dimorphism in ANGPTL4 expression was observed in humans. *2*) ANGPTL8 mRNA and protein levels in the liver and adipose tissue were higher in female than male mice. In humans, ANGPTL8 mRNA levels in adipose tissue were also higher in females than in males, whereas no sexual dimorphism in ANGPTL8 expression was observed in the liver. *3*) LPL protein levels in adipose tissue were higher in female than male *Angptl4*−/− mice, which was abolished by ANGPTL4 (over) expression. *4*) Sexual dimorphism was observed for the magnitude of the effect of ANGPTL4 on plasma TG and cholesterol—showing a more pronounced effect in male mice—which was likely related to the larger differences in ANGPTL4 levels among the three male *Angptl4* genotypes. *5*) Human genetic evidence indicates that ANGPTL4 inactivation has a similar effect on plasma TG in females and males, suggesting that ANGPTL4 is a suitable target for reducing plasma TG levels in both sexes.

In humans, consistent with the higher LPL activity in female adipose tissue (65, 66), *LPL* mRNA levels were higher in subcutaneous adipose tissue of females than males, which likely contributes to the lower fasting and post-prandial plasma TG levels in women. In contrast, in mice, *Lpl* mRNA levels were lower in the adipose tissue of females than males. In mice lacking ANGPTL4, LPL protein exhibited an opposite pattern, showing higher levels in female than male adipose tissue, which was abolished by ANGPTL4 (over) expression. The levels of LPL protein in mouse adipose tissue were not well mirrored by LPL activity. Hence, the effect of sex on LPL mRNA, protein, and activity are not fully consistent, suggesting that LPL is impacted by sex at multiple levels.

The effect of ANGPTL4 on LPL is more consistent. *Angptl4* genotype did not influence *Lpl* mRNA levels in adipose tissue, yet similarly regulated LPL protein and activity levels, giving rise to an inverse relationship between ANGPTL4 and LPL protein levels in adipose tissue. These findings are in agreement with the extensive data indicating that ANGPTL4 regulates LPL at the protein level, as previously summarized (11).

There is abundant evidence from cell-free enzymatic assays that ANGPTL8 alone does not influence LPL activity, yet impairs the ability of ANGPTL4 to inhibit LPL (67, 68). The inhibitory action of ANGPTL8 toward ANGPTL4 likely explains why the loss of ANGPTL8 in adipose tissue leads to higher plasma TG (50). Whether ANGPTL8 influences LPL protein levels in adipocytes has remained elusive. We find that the higher adipose LPL protein levels in female versus male *Angptl4*−/− mice are accompanied by higher ANGPTL8 protein levels, raising the possibility that ANGPTL8 may upregulate LPL protein levels and account for the higher adipose LPL content and activity in females than males. Considering that ANGPTL4 promotes LPL degradation in adipocytes—while initially, it was merely thought to be a non-competitive inhibitor of LPL—it could be hypothesized that ANGPTL8 may act oppositely to ANGPTL4 and reduce LPL degradation in cells (42, 57). Such a stabilizing action of ANGPTL8 could be weakened by ANGPTL4, leading to LPL unfolding and subsequent degradation. Further studies in ANGPTL8-deficient cells and/or mice are warranted to validate this hypothesis.

Previous in vitro studies indicated that ANGPTL8 lowers ANGPTL4 protein levels in adipocytes, possibly by promoting ANGPTL4 degradation (50). So far, it has not been studied if ANGPTL4 influences ANGPTL8 protein levels. In female mice, we found that both ANGPTL4 ablation and ANGPTL4 overexpression were associated with elevated adipose ANGPTL8 levels. Such a pattern was not observed in males, however, in which adipose ANGPTL8 levels seemed to be inversely correlated to ANGPTL4 levels. Hence, our data do not point towards a consistent effect of ANGPTL4 on ANGPTL8 levels in adipose tissue. Interestingly, in the livers of fasted male mice, an inverse association was found between ANGPTL8 and ANGPTL4 protein levels.

As indicated above, ANGPTL4 protein and mRNA levels were higher in the liver and adipose tissue of male mice than female mice. Nevertheless, we could not identify distinct effects of estradiol and testosterone on ANGPTL4 protein and mRNA levels in murine adipocytes and hepatocytes. Hence, the origin of the higher ANGPTL4 expression in male than female mice remains unclear. Opposite to ANGPTL4, ANGPTL8 protein and mRNA levels were higher in the liver and adipose tissue of female than male mice. *ANGPTL8* mRNA levels were also significantly higher in adipose tissue of women than men. Estradiol and testosterone similarly induced *ANGPTL8* mRNA levels in murine adipocytes, suggesting that the sexual dimorphism in ANGPTL8 expression is not due to distinct regulation by the sex hormones. Unfortunately, ANGPTL8 protein was not detectable in cultured murine adipocytes and hepatocytes, thus preventing the proper study of ANGPTL8 regulation by the sex hormones.

Even though in mice and humans, adipose ANGPTL8 mRNA and/or protein levels were significantly higher in females than in males, analysis of plasma protein data from the Icelandic cohort indicated that plasma ANGPTL8 levels were not significantly different between women and men. These data suggest a minimal contribution of adipose tissue to the plasma ANGPTL8 pool, which is consistent with data collected in mice (50). In humans, liver ANGPTL8 expression was not significantly different between females and males, likely explaining the lack of difference in plasma ANGPTL8 levels between the two sexes.

It is well established that PPARα upregulates hepatic ANGPTL4 expression during fasting (69). The notion that ANGPTL4 may also impact PPARα in the liver has not yet been raised. Here, we found that ANGPTL4 ablation led to the induction of PPARα target genes in the liver in the fasted state, including many genes involved in fatty acid oxidation. These data are consistent with data from the Fernandez-Hernando group, which found upregulation of fatty acid oxidation genes in the livers of hepatocyte-specific ANGPTL4-deficient mice (59). Evidence was presented that the induction of fatty acid oxidation is mediated by the dis-inhibition of hepatic lipase, leading to enhanced fatty acid uptake, ROS generation, and AMPK activation (59). Alternatively, it could be hypothesized that enhanced fatty acid uptake may cause increased PPARα activation (70, 71), which in turn triggers the upregulation of PPARα target genes involved in fatty acid oxidation. Similar to the mechanism in liver cells, deficiency of ANGPTL4 in endothelial cells was shown to promote lipase-mediated lipoprotein lipolysis, resulting in increased fatty acid uptake and oxidation (72). Intriguingly, the deficiency of ANGPTL3 in liver cells was also shown to increase fatty acid oxidation and upregulate PPARα target genes (73). Although it is tempting to attribute the above observations to the alleviation of lipase inhibition by ANGPTL3/ANGPTL4 deficiency—thereby resulting in increased fatty acid uptake—the actual evidence supporting hepatic and endothelial lipase inhibition by ANGPTL4 and hepatic lipase inhibition by ANGPTL3 is limited. Perhaps LPL, originating from extra-hepatic tissues, plays a larger role in the liver than generally envisioned. Regardless of the underlying mechanism, there is growing evidence that the deficiency of ANGPTL3 and ANGPTL4 leads to the activation of PPARα and fatty acid oxidation in the liver.

Our results support the therapeutic targeting of ANGPTL4 for reducing plasma lipid levels and cardiovascular risk in both sexes. Currently, strategies are being pursued to suppress the production of ANGPTL4 in the liver via antisense oligonucleotides and RNAi and the activity of ANGPTL4 in plasma via monoclonal antibodies. Previously, it was observed that genetic and antibody-mediated whole-body inactivation of ANGPTL4 in mice fed a diet rich in saturated fatty acids elicits severe and ultimately lethal chylous ascites and peritonitis, which is preceded by enlarged mesenteric lymph nodes, the presence of lipid-filled giant cells, and a massive acute phase response (30, 74). Liver-specific inactivation avoids this severe pathology in mice but may not lead to sufficient lowering of plasma TG (31, 59). Whole-body inactivation via monoclonal antibodies causes a more pronounced lowering of plasma TG yet has been shown to trigger lymphadenopathy in female monkeys (34). It should be noted, though, that lymphadenopathy appears to be disconnected from the severe chylous ascites and acute phase response (75). Importantly, in a recent targeted cis-pQTL mendelian randomization analysis (76), genetically lowered plasma ANGPTL4 levels via the ANGPTL4 p.E40K and p.Cys80 fs variants were not associated with any phenotypes related to lymphadenopathy and malabsorptive states. While these data do not entirely exclude any harmful effects of ANGPTL4 inactivation, they do mitigate safety concerns about the impact of whole-body inactivation of ANGPTL4 in humans. Targeted and combined liver- and adipose tissue-specific inactivation of ANGPTL4 is expected to sufficiently lower plasma TG to achieve a clinical benefit while avoiding lymphadenopathy. However, currently, there are no strategies to inactivate ANGPTL4 in human adipose tissue.

Our study also has limitations. In our studies, mice were fasted for 24 h, which roughly corresponds to the end of stage 3 of the five-stage model of fasting (77), characterized by the depletion of hepatic glycogen. In mice but not in humans, 24 h of fasting leads to substantial fat loss. The results of our studies might have been different if the mice had fasted for a shorter period. Another limitation is that we did not have access to ANGPTL8-deficient mice. As a result, we could not investigate if the higher adipose ANGPTL8 levels in females than males may contribute to their higher adipose LPL activity and lower plasma TG levels. Furthermore, we were unable to capture the ANGPLT8 protein in vitro using the available antibody. Consequently, we were unable to quantify the effects of sex hormones on levels of ANGPTL8 protein in mouse primary hepatocytes and adipocytes. Also, due to the absence of an antibody against human ANGPTL8, we were unable to verify at the protein level the increased *ANGPTL8* mRNA levels in human adipose tissue of females compared to males. Lastly, since we used whole-body *Angptl4*-Tg and *Angptl4*−/− mice to further explore the role of ANGPTL4 in the liver, we cannot rule out the possibility that the enhanced TG content and activation of PPARα in the fasted *Angptl4*−/− liver is due to the absence of ANGPTL4 in other tissues, such as adipose tissue.

In conclusion, male mice exhibit higher hepatic and adipose ANGPTL4 expression levels than female mice, as well as a more pronounced effect of genetic ANGPTL4 modulation on plasma lipids. By contrast, very limited sexual dimorphism in hepatic and adipose ANGPTL4 expression is observed in humans. Expression levels of ANGPTL8 in human and mouse adipose tissue are highly sexually dimorphic, showing higher levels in females than males, hinting at a possible role of ANGPTL8 in the sexually dimorphic LPL and plasma TG levels. Considering that the inactivation of ANGPTL4 effectively reduced plasma TG levels in female and male mice and that the human ANGPTL4 E40K loss-of-function variant was associated with similar plasma TG reductions in females and males, our results support the targeting of ANGPTL4 for reducing plasma lipid levels in both sexes.

## Data availability

The transcriptome data for the livers of fed and fasted *Angptl4*-Tg, WT, *Angptl4*-/mice have been submitted to Gene Expression Omnibus (accession number pending). All other data are contained within the manuscript or are already publicly available.

## Supplemental data

This article contains supplemental data (38).

## Conflicts of interest

Kersten is a paid consultant for Lipigon. The other author declares that she has no conflicts of interest with the contents of this article.
