# Supplementary material for: Characterization of sexual dimorphism in ANGPTL4 levels and function
Source: J Lipid Res. 2024 Feb 29;65(4):100526. doi: 10.1016/j.jlr.2024.100526 (PMC10973588; doi:10.1016/j.jlr.2024.100526)
Supplement: Supplemental data [file mmc1.pdf]

SUPPLEMENTAL DATA

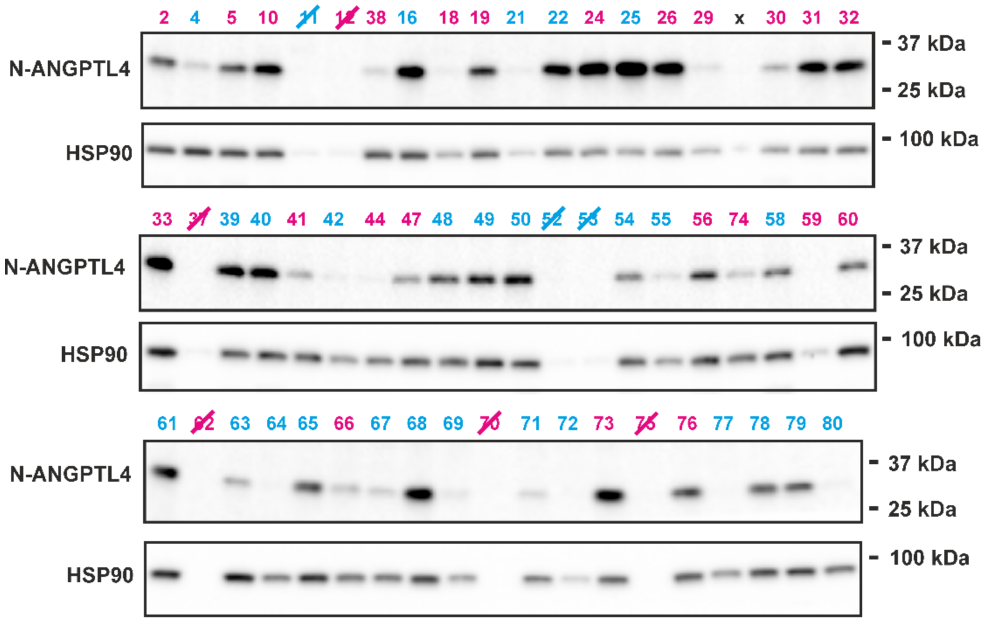

Supplemental figure 1. Protein levels of N-terminal ANGPTL4 in human liver. Human liver samples (10 µg) from the Mondial study were separated by SDS-PAGE. Western blots were probed with antibodies against ANGPTL4 (1:1000, #1187 Custom made) and HSP90 (1:2000, Cell Signalling). Samples from female subjects are indicated in pink, samples from male subjects in blue. Samples that are crossed out are not included in the quantification and comparative analysis due to weak signal.

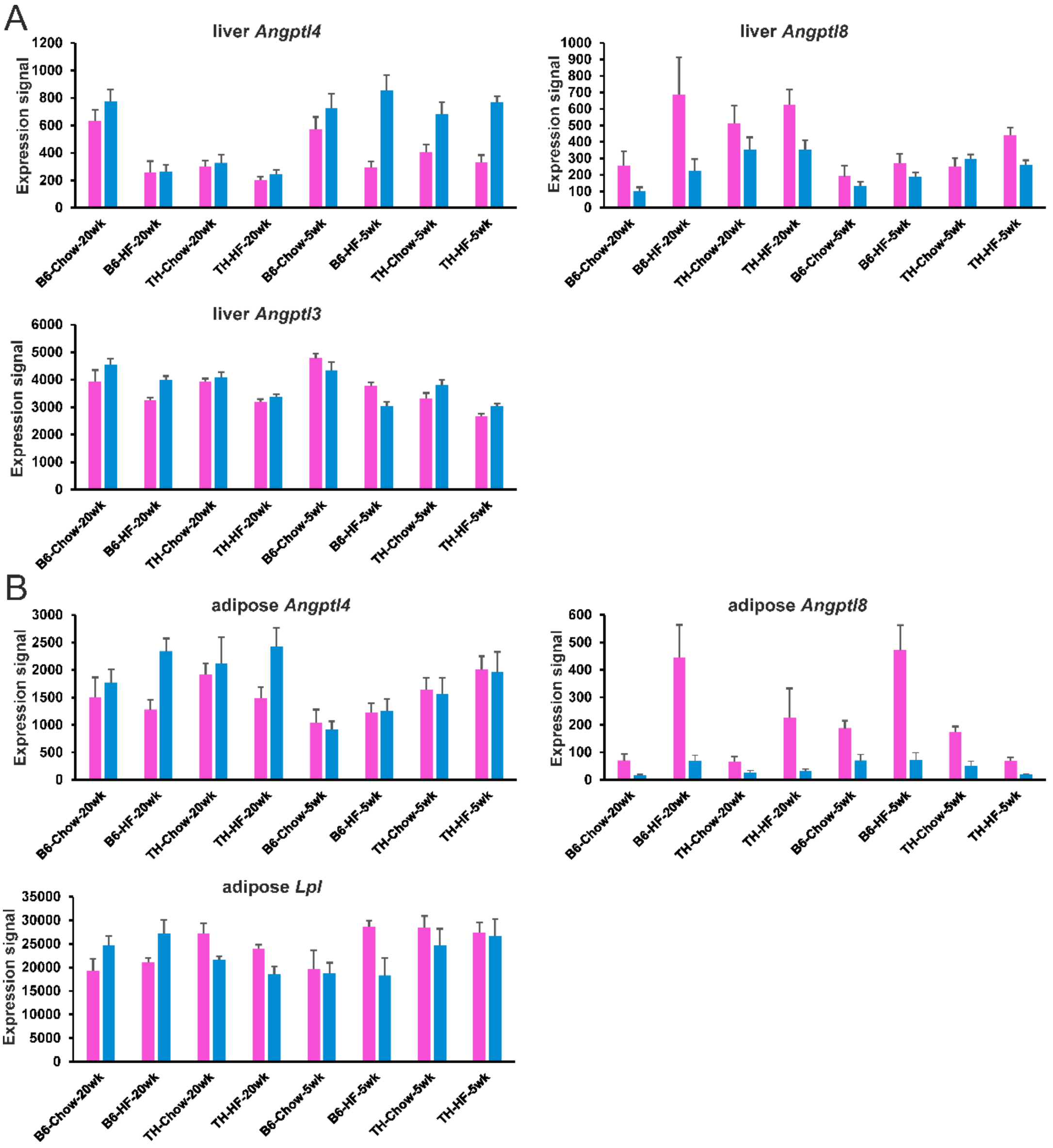

Supplemental figure 2. Comparative analysis of hepatic and adipose expression of Angiopoietin-like proteins between male (blue bars) and female (pink bars) mice. A) Relative mRNA levels of *Angptl3*, *Angptl4*, and *Angptl8* in livers of 5-week-old and 20-week-old female and male C57BL/6J (B6) and TALLYHO/Jng (TH) mice, based on bulk RNA-seq (GSE23401). B) Relative mRNA levels of *Angptl4*, *Angptl8*, and *Lpl* in adipose tissue of 5-week-old and 20-week-old female and male C57BL/6J (B6) and TALLYHO/Jng (TH) mice, based on bulk RNA-seq (GSE23401). N=6/group. Error bars represent SEM.

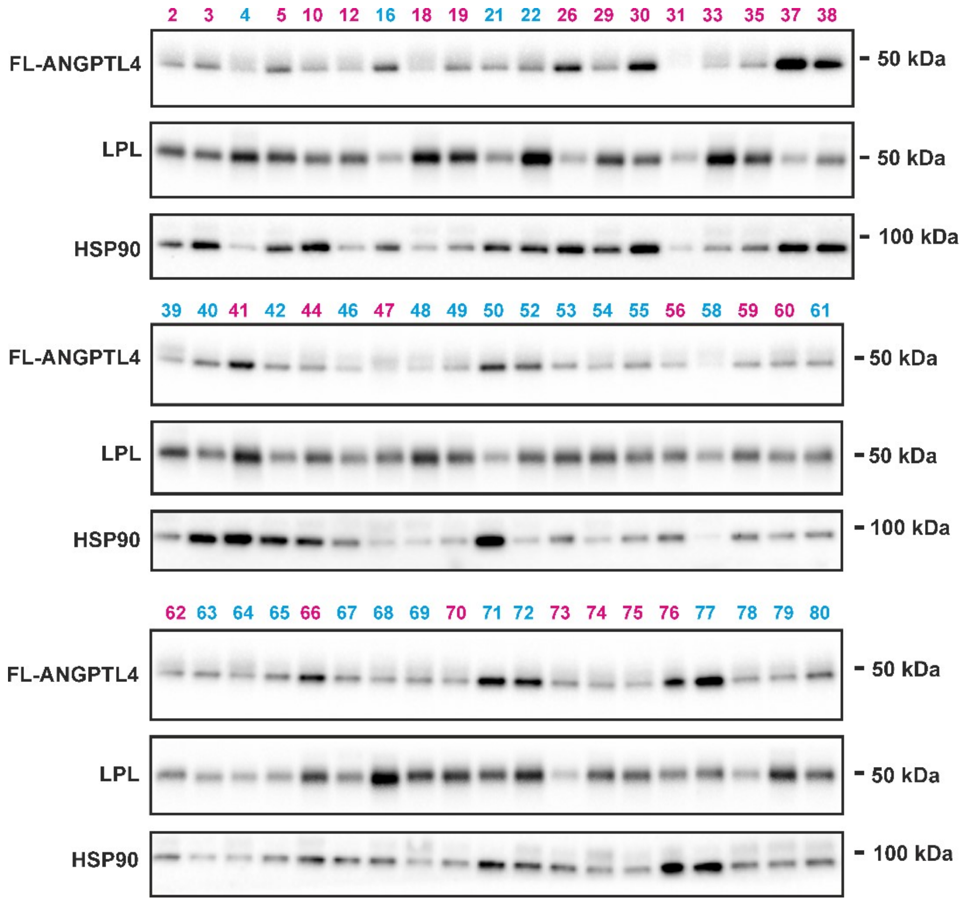

Supplemental figure 3. Protein levels of full-length ANGPTL4 and LPL in human subcutaneous adipose tissue. Human adipose tissue samples (5 µg) from the Mondial study were separated by SDS-PAGE. Western blots were probed with antibodies against LPL (1:750, Y-20 Santa Cruz), ANGPTL4 (1:1000, #1187 Custom made) and HSP90 (1:2000, Cell Signalling). Samples from female subjects are indicated in pink, samples from male subjects in blue. The LPL and ANGPTL4 Western blots were previously published without the specification male/female as a supplementary figure (1).

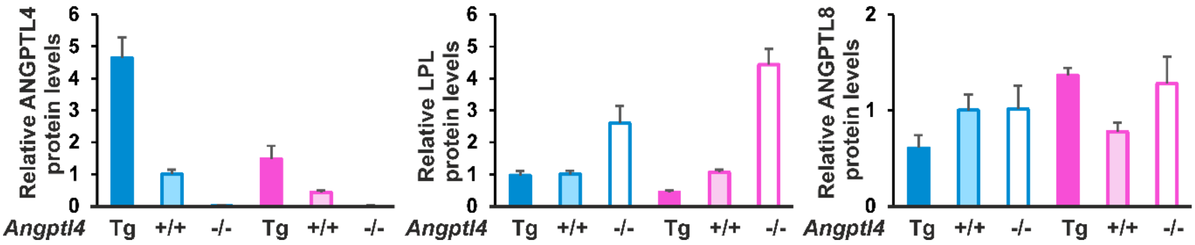

Supplemental figure 4. Quantitative densitometry of the Western blots in Figure 3E. Densitometry signals for HSP90 were used for normalization. N=4/group. Error bars represent SEM.

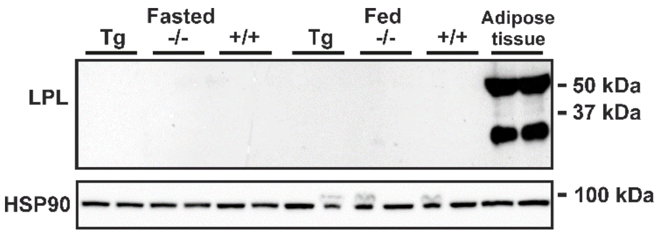

Supplemental figure 5. Protein levels of full-length LPL in livers of fed and 24h fasted male *Angptl4*-Tg, wildtype, and *Angptl4*-/- mice, as determined by Western blot. Adipose tissue of mice was used for comparison.

Reference

1. Dijk W, Schutte S, Aarts EO, Janssen IMC, Afman L, Kersten S. Regulation of angiopoietin-like 4 and lipoprotein lipase in human adipose tissue. J Clin Lipidol. 2018;12(3):773-83.
